# Long-Term Effects of a Cognitive Behavioral Conference Call Intervention on Depression in Non-Professional Caregivers

**DOI:** 10.3390/ijerph17228329

**Published:** 2020-11-11

**Authors:** Lara Lopez, Fernando L. Vázquez, Ángela J. Torres, Patricia Otero, Vanessa Blanco, Olga Díaz, Mario Páramo

**Affiliations:** 1 Department of Clinical Psychology and Psychobiology, University of Santiago de Compostela, 15782 Santiago de Compostela, Spain; fernandolino.vazquez@usc.es (F.L.V.); olga.diaz.fernandez@usc.es (O.D.); 2 Department of Psychiatry, Radiology and Public Health, University of Santiago de Compostela, 15782 Santiago de Compostela, Spain; angelajuana.torres@usc.es (Á.J.T.); mario.paramo@usc.es (M.P.); 3 Department of Psychology, University of A Coruña, 15008 A Coruña, Spain; patricia.otero.otero@udc.es; 4 Department of Evolutionary and Educational Psychology, University of Santiago de Compostela, 15782 Santiago de Compostela, Spain; vanessa.blanco@usc.es

**Keywords:** prevention, depression, non-professional caregiver, telephone, dismantling, long-term efficacy, cognitive–behavioral intervention

## Abstract

Recent evidence supports the efficacy of conference call cognitive–behavioral interventions in preventing depression in caregivers at post-intervention, but we do not know whether the results are sustained long term. The main objective of this study was to evaluate the long-term efficacy of a cognitive–behavioral intervention administered by telephone conference call in preventing depression in caregivers with elevated depressive symptoms, comparing all components of the intervention versus only the behavioral ones. A randomized controlled trial was conducted using a dismantling strategy. At total of 219 caregivers were randomly assigned to a cognitive–behavioral conference call intervention (CBCC; *n* = 69), a behavioral-activation conference call intervention (BACC; *n* = 70), or a usual care control group (CG, *n* = 80). Information was collected on depressive symptoms and depression at pre-intervention and at 1, 3, 6, 12, and 36 months post-intervention. At 36 months, there was a reduction in depressive symptoms (*p* < 0.001) and a lower incidence of major depressive episodes in both the CBCC and BACC groups compared to CG (8.7%, 8.6%, and 33.7%, respectively). The results show that a conference call intervention was effective in the long term to prevent depression in caregivers and that the behavioral-activation component was comparable to the complete cognitive–behavioral protocol.

## 1. Introduction

Currently, more than 10% of the adult population in the countries of the Organization for Economic Cooperation and Development (OECD; [1]) and 34.3% of the European population [2] perform non-professional care tasks. This work has serious repercussions on caregivers’ mental health, with depression being one of the most significant. It is estimated that this disorder affects 8.9% of the caregiver population [3], with between 34% and 40% exhibiting a high level of depressive symptoms [4,5].

Depression negatively impacts non-professional caregivers’ quality of life (e.g., [6]) and health (e.g., [7]), and represents a potential risk factor for other negative outcomes, such as Post-Traumatic Stress Disorder (PTSD; e.g., [8]). It also affects the quality of the care provided (e.g., [9]) and is associated with an increased probability of institutionalization for the care recipient [10]. The accumulated evidence indicates that sub-syndromic depressive symptoms are one of the most powerful predictors of depression [11,12,13].

Taking all of this into account, indicated depression prevention programs for caregivers could be very useful because they are aimed at preventing depressive episodes among those who exhibit a high level of symptoms but who do not yet meet the diagnostic criteria for major depression. Indicated prevention interventions for depression have been shown to be efficacious [14] and cost-effective [15], and therefore the administration of this type of approach among the caregiver population is recommended [16].

Nevertheless, only two randomized controlled trials (RCT) have evaluated face-to-face indicated prevention interventions for depression aimed at non-professional caregivers. These interventions showed efficacy in the short term [17,18] and long term [19,20,21]. However, face-to-face interventions present a series of barriers to participation, such as a shortage of mental health services (especially in rural areas), transportation difficulties, cost, and mental health stigma. Furthermore, caregivers experience added difficulties, such as a lack of time or not having someone to replace them in providing care during their absence.

A feasible alternative to overcome these obstacles is the administration of interventions over the phone. One meta-analysis found that psychological interventions for mood disorders delivered over the phone achieved significant reductions in depressive symptoms, with a small to moderate effect size (Cohen’s *d* = −0.42; [22]). Regarding studies specifically focused on non-professional caregivers, RCTs that evaluated the efficacy of telephone interventions for depressive symptoms have found contradictory results. While some studies found the interventions to be effective (e.g., [23,24,25,26]), others found no significant difference between the intervention and control groups (e.g., [27,28,29,30]). Still, others found partial support for the telephone interventions that were limited to certain subgroups of caregivers (e.g., [31,32]) or at certain follow-up points (e.g., [33,34,35,36]) regarding depressive symptoms. However, some studies had significant results in other outcomes, such as a high percentage of complete or partial attainment [33], an improvement in well-being [34,35,36], coping with the care situation [34,36], perceived health [34], physical health [35,36], quality of life [35], the behavior of the care recipient [36], and a reduction in physical complaints [34,35].

Furthermore, despite the importance of analyzing the long-term effects of interventions (e.g., [37,38]), only three RCTs have performed follow-up evaluations [35,36,39]. The results for reported depressive symptoms of these long-term evaluations were not very encouraging. Donath et al. [39] analyzed the long term data of the cluster-randomized controlled trial by Berhndt et al. [32] in which 453 caregivers received a telephone advice intervention (*n* = 263) or usual care (*n* = 190). There were no significant differences between the two groups at the 12-month follow-up, although secondary analyses found a moderate effect size (Cohen’s *d* = 0.52) for caregivers of people with mild dementia. Furthermore, in two follow-up analyses of their 2011 study, Wilz et al. [34,35] examined the long-term effects of a cognitive–behavioral intervention (*n* = 50), a progressive muscle relaxation control group (*n* = 53), or a usual care control group (*n* = 50). At the six-month follow-up, participants who had received the cognitive-behavioral intervention reported significantly fewer depressive symptoms compared to the progressive muscle relaxation group, with a small effect size (Cohen’s *d* = 0.26) [34]. At the two-year follow-up [35], only the cognitive–behavioral intervention and usual care control group were compared, and no significant differences were found in depressive symptoms. Finally, Wilz et al. [36] conducted a RCT with 273 caregivers, who were assigned to a cognitive–behavioral therapy intervention group (*n* = 139) or a usual care control group (*n* = 134); significant differences in depressive symptomatology were not maintained at 6 months post-intervention. These last two studies [35,36], however, found effects from the interventions in other important outcome variables, such as emotional well-being, and, in the subgroup of caregivers still caring at home, perceived health, bodily complaints, and quality of life at the two-year follow-up [35]. Likewise, significant improvements were also observed in well-being, physical health symptoms, coping and behavior of the care recipient at the six-month follow-up [36].

An additional limitation is that we have only one RCT focusing on indicated prevention of depression delivered on the phone in this population [40]. In this study, the intervention was effective in reducing both depressive symptoms and the appearance of new depressive episodes post-intervention. Furthermore, the recommendations to identify the elements of an intervention that are necessary and sufficient to achieve therapeutic change [38,41], and to conduct dismantling studies for interventions of proven efficacy [42,43], led to the study of a complete cognitive–behavioral intervention versus an intervention using only the behavioral-activation component [40]. Specifically, 219 caregivers with elevated depressive symptoms were randomly assigned to a cognitive–behavioral conference call intervention (CBCC; *n* = 69), a behavioral-activation conference call intervention (BACC; *n* = 70), or a usual care control group (CG, *n* = 80) (for further detail regarding the contents of the interventions, please see the Materials and Methods section). At post-intervention, both interventions reduced depressive symptoms compared to the control group (*d* = 1.16 for CBCC and 1.29 for BACC), and prevented depression compared to the control group (1.5% incidence of major depressive episodes for CBCC, 1.4% for BACC, and 8.8% for CG). The BACC intervention was as effective as the complete CBCC. However, we do not yet know whether this treatment effect maintained long term efficacy.

Likewise, we also do not know what caregiver sociodemographic variables and care situation variables would allow for identification of caregivers who are likely to benefit from these telephone preventive interventions (i.e., moderators) or the mechanisms that account for the effects found on depressive symptoms after administering the interventions (i.e., mediators). Analysis of this latter question is essential because dismantling the intervention allows us to identify the components of the program that are responsible for the results, but it does not provide information on the mechanisms that explain how these changes take place [44]. In a now-classic study, Jacobson et al. [45] found that the initial change in attributional style mediated the change in depressive symptoms in a behavioral activation intervention (BA), but not in a multicomponent cognitive–behavioral intervention (CB) based on behavioral activation, modification of negative automatic thoughts, and the modification of central beliefs, and the change in the frequency of enjoyable activities mediated the change in CB (but not in BA). More specifically, the mediation effects of this type of intervention in relation to the reduction of depressive symptoms are unknown. However, Wilz et al. [36] and Töpfer and Wilz [46] identified the use of resources related to well-being and coping as mediators of the effects of the applied cognitive behavioral intervention on quality of life. Therefore, more knowledge is needed on the mechanisms of change that operate in cognitive–behavioral interventions for the reduction of depressive symptoms, especially those aimed at caregivers.

The main objective of this study was to evaluate the long-term efficacy (up to 36 months) of a complete cognitive–behavioral intervention versus the behavioral activation component alone administered via telephone conference call to prevent depression in caregivers with high depressive symptoms. We expected to see significant differences in depressive symptomatology and the incidence of major depressive episodes between the two interventions and the control group at follow-up time points of up to 36 months. The secondary objective was to analyze the moderators and mediators of the effects of both interventions. We expected that the sociodemographic characteristics and the care situation would be moderators of the effect of the interventions and that changes in negative cognitions and the level of reinforcement would act as mediators in the intervention effects.

## 2. Materials and Methods 

The methodology of the current study has been previously published [40,47], though key methodological aspects are highlighted below.

### 2.1. Participants and Procedure

An RCT (trial code NCT02292394) was conducted between December 2014 and December 2015 to examine the components of the cognitive–behavioral intervention using a dismantling strategy. The behavioral-activation component of the intervention was examined by eliminating the cognitive component in the intervention in one of the comparison groups [48]. The study sample was recruited through consecutive enrollment from the official registry of informal caregivers identified by the Government of the Autonomous Community of Galicia, a region in northwestern Spain with 2,730,337 inhabitants, among those providing care for people classified as significantly dependent (scoring at least 75 out of 100 points in the dependency assessment scale administered by members of the dependency assessment team from the applicable government agency). Participants were contacted by mail to invite them to participate and asked to return a sealed postcard if they did not want to be contacted again. Those who did not return the postcard were contacted by phone and offered a brief description of the study. Those who were interested participated in an initial screening to assess the presence of depressive symptoms and their history of depressive episodes. Those who met the initial selection criteria were invited to participate in a more extensive evaluation that determined whether they met the eligibility criteria. The inclusion criteria were: (a) being an informal caregiver for a person whose situation of dependency was determined by technical specialists of the government of the Autonomous Community of Galicia; (b) presenting with a high risk of depression (>16 on the Spanish version of the Depression Scale of the Center for Epidemiological Studies (CES-D; [49]); (c) not meeting the Diagnostic and Statistical Manual of Mental Disorders, 5th ed. (DSM-5) diagnostic criteria for a current a major depressive episode [50]; (d) not having a history of major depression; (e) having a phone; and (f) committing to participate in all evaluations. 

The exclusion criteria were: (a) having received psychological or pharmacological treatment in the last two months; (b) presenting other disorders that could act as confounding variables (dysthymia, bipolar disorders I and II, anorexia, psychotic disorders, dependence on alcohol or other substances, panic disorder, obsessive–compulsive disorder, somatization disorder, hypochondria, undifferentiated somatoform disorder); (c) having serious psychological or medical disorders that require immediate intervention (e.g., suicidal ideation) or that would make it impossible to complete the study (e.g., significant cognitive impairment, severe hearing impairment); (d) being involved in another study; (e) anticipating a change of residence or institutionalization of the dependent person; and (f) the dependent person having a severe or terminal prognosis for the next 14 months.

**Figure 1:**
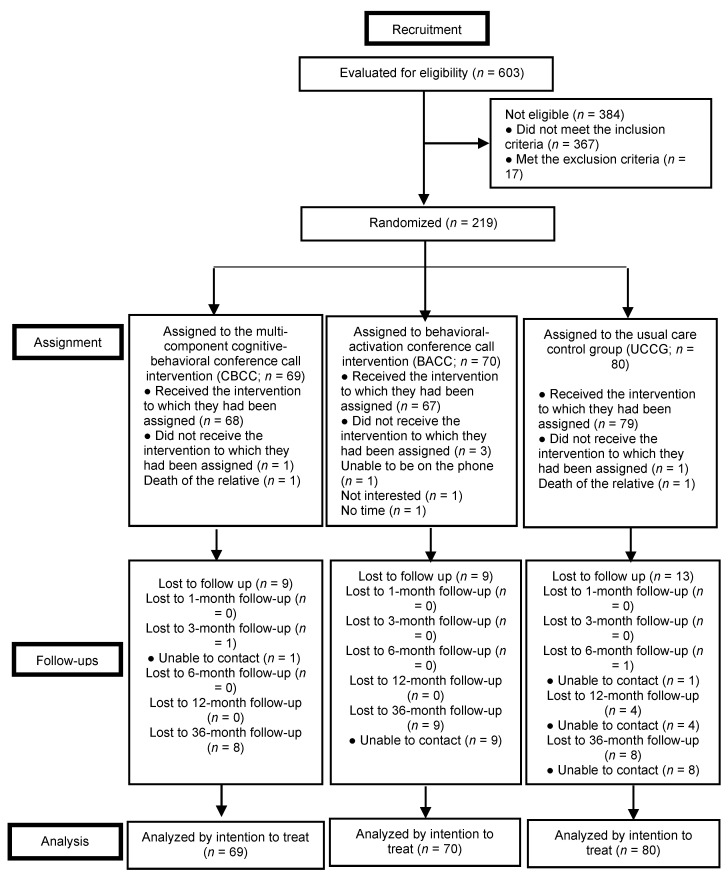
Flowchart.

As Figure 1 shows, the final sample consisted of 219 caregivers who were randomly assigned to one of the three experimental groups by an independent statistician. To minimize the loss of participants, the strategies for maximizing follow-up and adherence in clinical trials recommended by Grady, Cummings, and Hulley [51] were followed, such as selecting participants who are likely to adhere to the intervention (excluding probable losses), making the intervention easy (using language adapted to the participants’ level, scheduling simple tasks between sessions applicable to their daily lives), using appointment times that are convenient for participants (scheduling sessions according to their availability, including nights and weekends, speeding up the times between evaluation and intervention to avoid a waiting time, having coordinated and organized intervention personnel), and obtaining different channels of communication with the participants (including address, telephone numbers and email).

The study was conducted following the principles of the Declaration of Helsinki [52] and obtained the approval of the Bioethics Committee of the University of Santiago de Compostela (Spain). Participation was entirely voluntary, and there were no financial or other incentives. All participants signed an informed consent form.

### 2.2. Interventions and Control Group

Table 1 contains the contents of each session of the programs. Both interventions were developed and manualized before the start of administration. They both consisted of five weekly sessions of 90 min each, in groups of approximately five participants. 

The cognitive–behavioral conference call intervention (CBCC) group received a brief cognitive–behavioral intervention based on Lewinsohn, Hoberman, Teri, and Hautzinger’s [53] multifactor integrative model of depression. This intervention was adapted from an indicated depression prevention program that had previously demonstrated short- and long-term efficacy in a randomized controlled trial administered in a face-to-face group format [18,21]. Session 1 explained the concept of depression and the need to actively cope with depressive symptoms. Participants were trained in diaphragmatic breathing, mood monitoring, and self-reinforcement techniques. Session 2 focused on how enjoyable activities affect mood and developing a plan to introduce enjoyable activities into participants’ daily lives through behavioral contracts. Session 3 addressed how thoughts affect mood and participants were trained in techniques to manage thoughts. Session 4 addressed how social contacts affect mood and participants were trained in assertive communication and how to increase social contacts. Session 5 reviewed everything learned and addressed relapse prevention.

The behavioral-activation conference call (BACC) group also received an intervention adapted from the program developed by Vázquez et al. [18,21], although it focused exclusively on the behavioral-activation component of the intervention. Session 1 addressed the concept of depression and the need to actively cope with depressive symptoms, and participants were trained in diaphragmatic breathing, mood monitoring, and self-reinforcement strategies. Session 2 explained how enjoyable activities affect mood, and a plan was developed to introduce enjoyable activities that can be done at home. Session 3 focused on increasing enjoyable activities outside the home. Session 4 addressed how social contacts affect mood, and participants developed a plan to increase them throughout the week. Session 5 reviewed everything learned and addressed relapse prevention.

The main difference between both interventions is that CBCC addressed how thoughts affect mood and participants were trained in techniques to manage thoughts (Session 3) and assertive communication (Session 4). However, the behavioral-activation conference call (BACC) intervention did not address these issues, and an equivalent amount of time was spent in specifically addressing how to increase enjoyable activities (at home, outside the home, and social). 

The two interventions were administered through a conference call system, in a group format. The adaptations consisted of adjustments to make the change from the face-to-face format to the conference call system (see [40,46] for more details). Four psychologists previously trained by two therapists with more than 25 years of experience in psychological intervention in the area of depression implemented the interventions: two for the CBCC intervention and two for the BACC intervention. One of the clinicians who participated in the training provided weekly supervision. After the intervention, there were no significant differences in depressive symptoms among participants depending on the two therapists who implemented the CBCC intervention (*t* (68) = 0.645, *p* = 0.521) or in the incidence of depressive episodes (*p* = 0.225). There were also no differences in depressive symptoms among participants after the intervention between the two therapists who administered the BACC intervention (*t* (67) = 0.617, *p* = 0.539) or in the incidence of depression (*p* = 0.350). The intervention sessions were recorded with the prior authorization of all the participants to assess the therapists’ adherence to the protocol. A random selection of 20% of the recordings from each therapist were chosen (11 sessions per therapist, 44 in total). Two clinicians (one of the clinicians who participated in therapist training and one independent senior clinician; both had more than 25 years of experience in psychological intervention in the area of depression) evaluated the recordings to estimate adherence to the established protocols. The two clinicians noted the number of topics implemented by the therapists compared to the total number of core elements in the treatment manuals (26 elements for the CBCC intervention, 25 for the BACC intervention). Protocol adherence resulted in a mean of 24.0 (SD = 0.4) of topics implemented in the CBCC group (92.3%) and 23.5 (SD = 0.7) in the BACC group (94.0%), indicating that the main elements of the protocol had been administered. The intraclass correlation coefficient for the adherence scores was 0.96 for the CBCC program and 0.99 for the BACC. 

The control group (CG) was a usual-care condition. The participants assigned to this group did not receive any psychoeducational intervention or material but were free to make use of any medical or psychological treatment (public or private) available in their community to address their depressive symptoms. Their use of such services was noted. A total of 31 caregivers (38.8%) in the control group received psychological or psychiatric care during the study period. Participants in this group were assessed at the same times as participants in the intervention groups.

### 2.3. Evaluation Instruments

The evaluations were conducted at the pre-intervention and post-intervention time points and at 1, 3, 6, 12, and 36-month follow-ups. Data were collected through self-administered instruments sent to participants via email or postal mail, and by instruments administered by interviewers over the phone. The interviewers, who were not part of the study staff, were trained specifically for this study and were unaware of the purpose of the study, the interventions administered, and the participants’ group assignments. 

Depressive symptomatology was evaluated using the CES-D ([54]; Spanish version from Vázquez et al. [49]), a self-administered 20-item instrument with a Cronbach alpha of 0.89. The diagnosis of major depressive episodes was made using the Structured Clinical Interview from the DSM-5, Clinical Version (SCID-5-CV [55]), a semi-structured interview that provides the most common diagnoses for clinical practice according to the DSM-5 and which must be administered by a clinician. Cronbach’s alpha for all disorders was >0.80 [56]. Perceived environmental reinforcement, negative automatic thoughts, and social contacts were also evaluated. Response-contingent positive environmental reinforcement was evaluated using the Environmental Reward Observation Scale (EROS [57]; Spanish version from Barraca and Pérez-Álvarez [58]), a 10-item self-reported scale with a Cronbach’s alpha of 0.86. Negative automatic thoughts were evaluated using the Automatic Thoughts Questionnaire (ATQ [59]; Spanish version from Otero, Vázquez, Blanco, and Torres [60]), a 30-item administered instrument with a Cronbach alpha of 0.96. Social contacts during the last week were evaluated using the Social Contacts Record (SOC), which had already been prepared and used in a previous study [18,21].

### 2.4. Statistical Analysis

The analyses were conducted using the SPSS statistical package (version 24.0, IBM Corp., Armonk, NY, USA) and the freeware program R (version 3.0.3, R Foundation for Statistical Computing, Vienna, Austria) [61], according to the intention-to-treat principle. The missing values of participants who dropped out of the study were imputed following the multiple imputation method, with the EMB algorithm [62] from the *Amelia II* program [63]. Fifteen imputations were made. An appropriate imputation model was determined for each of the variables (one that does not exceed 10% of ranges that exclude the straight line y = x) by overimputation. The methodology proposed by Jolani et al. [64] for multilevel imputation of binary data was used to impute the missing values for the major depressive episode variable. The parameters from the different models and tests were combined using the *mice* and *miceadds*, *mitml* and *mediation* packages for R [65,66,67,68].

Depressive symptoms among the study conditions and between the various follow-up points were compared using linear mixed models (LMM, [69,70]). The χ^2^ statistic was inferred from the results of the analysis of variance (ANOVA) table for the model, based on the 15 imputed databases, to calculate the D2 statistic [71]. The mixed models were fitted using the Ime4 package [72]. In the a posteriori contrasts, in addition to the Bonferroni correction, we calculated the *p*-values resulting from the correction of Holm–Bonferroni and of Benjamini and Yekutieli [73]. Effect sizes were calculated taking into account that the estimated models are mixed models, and effect sizes *d* = 0.2 were considered small, *d* = 0.5 as moderate, and *d* = 0.8 as large [74]. The emmeans [75] and multcomp [76] packages were used to fit the *p*-values for the different contrasts and to obtain the effect measurements. 

The incidence of major depressive episodes at the 36-month follow-up was analyzed for the three groups and Cox regression was used to determine whether there were significant differences between the groups. In addition, relative risk (RR) and number of patients needed to treat (NNT) were calculated according to the formulas proposed by Guyatt, Sackett, and Cook [77]. The survival function (at each time point and for each intervention group) was estimated using the Andersen-Gill model [78], an extension of the Cox model for recurrent events.

Finally, descriptive statistics were used to analyze the impact of death of the care recipients for participants at the 36-month follow-up (*n* = 183); the percentage of deaths was compared among the three groups using a Chi-square test. To analyze the potential effect of the death of the care recipient at the 36-month follow-up, the LMM model [69,70] and the Andersen–Gill model [78] were replicated, including the death of the relative as an adjustment variable.

#### Moderation and Mediation Analyses

Moderation and mediation were analyzed separately for the CBCC and BACC groups. All the moderation and mediation analyses were conducted on the 15 databases obtained after imputation, combining the results of the different models according to Rubin’s rules [79]. The variables were centered following the recommendations of Kraemer and Blasey [80].

An analysis was conducted to determine whether the change in depressive symptoms between pre-intervention and the 12-month follow-up was moderated by the sociodemographic, care, or clinical variables evaluated at baseline (see Table 2). The 12-month follow-up was chosen as the time point to analyze moderation because it represents the high point of indicated prevention. This is because, according to a recent meta-analysis [81], the effects of interventions increase between 6 and 12 months, and studies using active comparison groups only found significant differences between groups at that follow-up. An approximation based on the linear regression model proposed by Baron and Kenny [82] was used to evaluate the potential moderating effect of the variables, adjusting for baseline depressive symptoms. A model in which the response is the change in depressive symptoms between 12 months and baseline was also evaluated. The interaction was evaluated comparing the restricted model (with the same variables but without interaction) versus the complete model, as proposed by Li, Raghunathan, and Rubin [83]. Terms of significant interaction provided evidence of a moderating effect on the relationship between experimental condition and change in depressive symptomatology.

In order to analyze potential mediators, the differences in depressive symptoms between the pre- and post-intervention time points were used as a dependent variable (Y), the intervention as an independent variable (X), and the difference in response-contingent positive environmental reinforcement, negative automatic thoughts, and social contacts as potential mediators (M). A simple mediation analysis without covariates or interaction was conducted. Three regression equations were fitted: Y = α + cX + ϵ (association between the independent variable and the dependent variable; although the classical approach considers a significant effect to be necessary, more current approaches do not have this requirement [84]); M = α + aX + ϵ (association between the independent variable and the mediator); Y = α + bM + c′X + ϵ (association between the mediator and the dependent variable, controlling for the independent variable). Following Hayes’s [85] recommendations, the indirect effect of mediation was estimated as c − c′ = ab. The mean direct effect was also estimated controlling for the mediator, total effect (c = c′ + ab), and the proportion of the effect that was mediated (ab/c). The significance of all these indicators was obtained from their bootstrap-estimated confidence intervals. Mediation was considered complete if the effect of X on Y, controlling for M, was 0 (c′ = 0); otherwise, mediation was deemed to be partial.

## 3. Results

### 3.1. Characteristics of the Sample

This study evaluated 603 caregivers; 236 met the eligibility criteria and 17 (7.2%) declined to participate. This left a final sample of 219 caregivers. Table 2 shows the sociodemographic, clinical, and care-situation characteristics of the sample (*n* = 219). Women accounted for 90.9% of the sample, with a mean age of 54.0 years (SD = 10.8). There were no significant differences between the groups for any of the sociodemographic, care situation, and clinical variables at baseline.

At the 36-month follow-up, 183 participants (83.6%) had completed all the evaluations. There were 36 dropouts, 10 (14.5%) from the CBCC group, 12 (17.1%) from the BACC group, and 14 (17.5%) from the CG group. The results of Fisher’s Exact Test (*p* = 0.044) revealed differences between those who dropped out and those who did not in terms of their main economic activity: those who did not have paid employment or who were retired dropped out at a higher rate. No significant differences were found among the rest of the sociodemographic, care situation, or clinical variables.

### 3.2. Changes in Depressive Symptoms over Time

The estimated mean CESD scores (and standard errors) for each of the groups at the different evaluated time points are shown in Table 3. At the pre-intervention time point, the mean scores for depressive symptoms ranged from 22.3 to 23.1. At the post-intervention time point, these scores were reduced significantly in the intervention groups and slightly in the control group, a trend that was maintained until the 36-month follow-up. 

**Figure 2:**
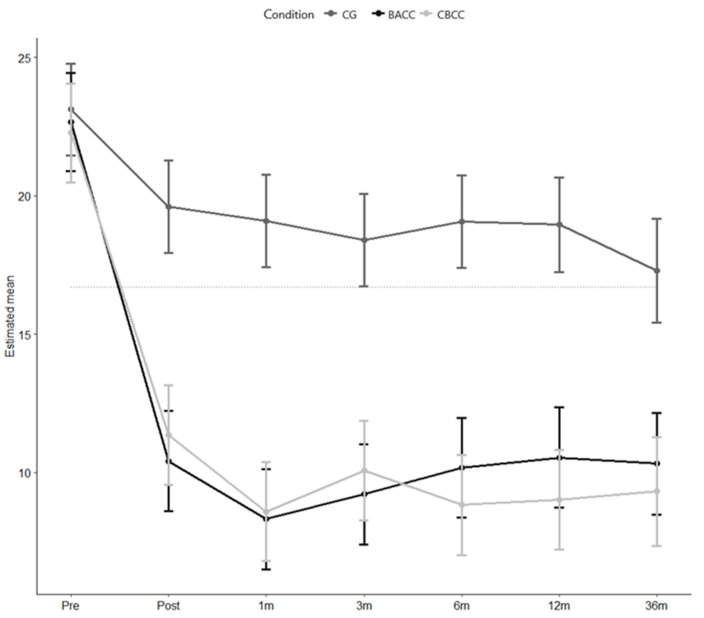
Estimated means at each time point for each group.

Figure 2 shows the (marginal) means estimated by the model for depressive symptoms for each evaluation time point, and for each group, through the 36-month follow-up. 

This figure shows how depressive symptoms decreased sharply (11 points) in the CBCC group between the pre-intervention and post-intervention time points—a difference that rose to 13.7 points at the 1-month follow-up, 12.2 points at the 3-month follow-up, 13.5 points at the 6-month follow-up, 13.3 points at the 12-month follow-up, and 13.0 points at the 36-month follow-up. Likewise, the reduction of depressive symptoms in the BACC group was also pronounced between the pre-intervention and post-intervention time points (13.3 points). This difference increased to 15.4 points at the 1-month follow-up, and was 14.5 points at the 3-month follow-up, 13.5 at the 6-month follow-up, 13.2 at the 12-month follow-up, and 13.4 at the 36-month follow-up. However, in the CG group, depressive symptoms were only reduced 3.5 points between the pre-intervention and post-intervention time points. This difference increased to 4 points at the 1-month follow-up, to 4.7 points at the 3-month follow-up, 4.0 points at the 6-month follow-up, and 4.1 at the 12-month follow-up, achieving a reduction in the estimated mean score for depressive symptoms of 5.8 points compared to the pre-intervention score at the 36-month follow-up.

Significant effects were found for group (D2 statistic: F (2, 8328.78) = 49.148, *p* < 0.001) and time (D2 statistic: F (6, 613.96) = 86.174, *p* < 0.001). All three groups showed statistically significant improvements at the post-intervention time point and in at the 1, 3-, 6-, 12-, and 36-month follow-ups compared to pre-intervention values (see Table 3). 

The group x time interaction was significant (D2 statistic: F (12, 6521.13) = 9.423, *p* < 0.001). Significant differences were found between the CBCC and BACC groups versus the CG control group at the post-intervention time point, which were maintained at the 1-, 3-, 6-, 12-, and 36-month follow-ups (see Table 4). No significant differences were found between the CBCC and BACC groups at any measurement point. 

### 3.3. Change in the Appearance of New Episodes of Major Depressive Episodes over Time

At the 36-month follow-up, 5 of the 69 (7.2%) participants in the CBCC group, 6 of the 70 (8.6%) participants in the BACC group, and 26 of the 80 (32.5%) in the CG presented with a major depressive episode. After imputing the missing values, at the 36-month follow-up, 6 of the 69 (8.7%) participants in the CBCC group, 6 of the 70 (8.6%) participants in the BACC group, and 27 of the 80 (33.7%) in the CG presented with a major depressive episode. Regarding the RR and NTT indicators, at 36 months of follow-up, the RR for CBCC was 8.7/33.8 = 0.26 (95% CI 0.11, 0.59) and the NTT was ≈4; the RR for BACC was 8.6/33.8 = 0.25 (95% CI 0.11, 0.58) and the NTT was ≈4.

**Figure 3:**
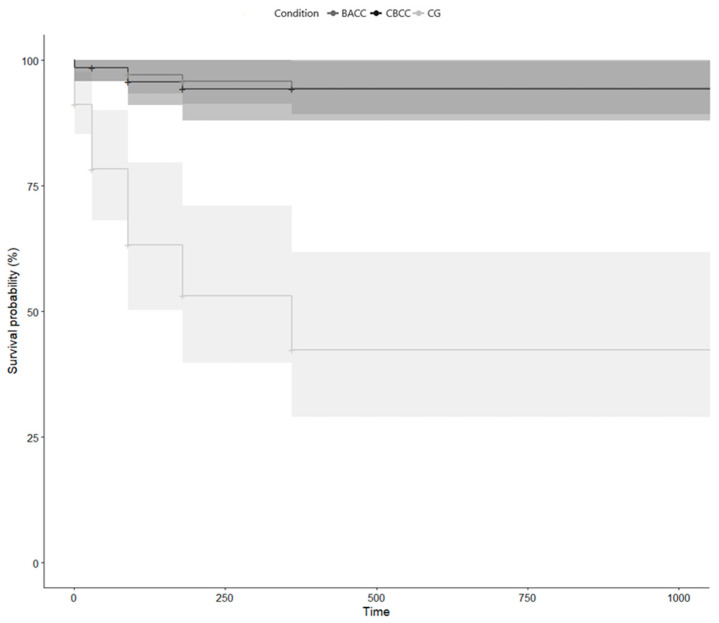
Cumulative survival for recurring events for the different experimental conditions.

Concerning the change in depressive episodes over 36 months, Figure 3 shows a survival analysis for recurring events over time for the incidence of major depressive episodes in the three groups. In the CBCC group, 1 major depressive episode was recorded at the post-intervention time point, 0 at the 1-month follow-up, 2 at the 3-month follow-up, 1 at the 6-month follow-up, 0 at the 12-month follow-up, and 3 at the 36-month follow-up (mean time: 1036 days). In the BACC group, 1 major depressive episode was recorded at the post-intervention time point, 0 at the 1-month follow-up, 1 at the 3-month follow-up, 1 at the 6-month follow-up, 1 at the 12-month follow-up, and 3 at the 36-month follow-up (mean time: 1027 days). In the CG group, 7 major depressive episodes were recorded at the post-intervention time point, 11 at the 1-month follow-up, 15 at the 3-month follow-up, 12 at the 6-month follow-up, 16 at the 12-month follow-up, and 16 at the 36-month follow-up (mean time: 774 days). Survival distributions for the three groups were significantly different (weighted χ^2^ = 30.25, *p* < 0.001). The Hazard ratio (HR) (weighted) was lower for the CBCC group with respect to the control group (HR = 0.11, 95% CI = 0.03–0.29) and for the BACC group with respect to the control group (HR = 0.14, 95% CI = 0.06–0.36). There was no difference in survival between the two interventions (HR = 0.70, 95% CI = 0.20, 2.51; *p* = 0.585).

### 3.4. Impact of Death of Care Recipient on Depressive Symptoms and Incidence of Depression

At the 36-month follow-up, 35.5% of care recipients (*n* = 65) had died: 19 of 54 care recipients in the CBCC group (35.2%), 20 of 60 care recipients in the BACC group (33.3%), and 26 of the 69 in the SC group (37.7%). There was no statistically significant difference in the number of deaths among the three groups, χ^2^ (2) = 0.269, *p* = 0.874. The effect of the interventions on the severity of depressive symptoms was statistically significant when this covariate was included in the mixed linear regression model (LMM; (χ^2^ (2) = 24,493, *p* < 0.001). The effect of the interventions on the incidence of depression, including the death of the dependent person as a covariate in the Andersen–Gill model, resulted in a lower (weighted) HR for the CBCC group with respect to CG (HR = 0.10, 95% CI = 0.03–0.29, *p* < 0.001) and for the BACC group with respect to CG (HR = 0.15, 95% CI = 0.06–0.37, *p* < 0.001). No differences in survival were found between the two interventions (HR = 0.66, 95% CI = 0.19, 2.34; *p* = 0.524).

### 3.5. Moderators and Mediators of Change in Depressive Symptoms

The moderation and mediation analyses were performed on the difference in scores for depressive symptoms between the pre-intervention time point and the 12-month follow-up.

**Figure 4:**
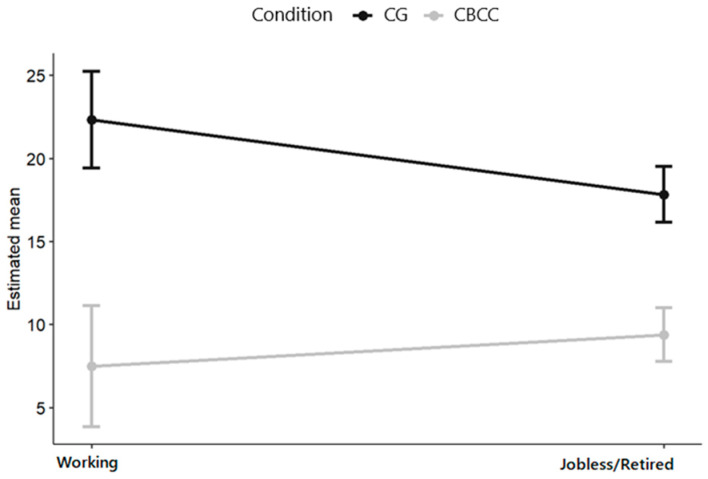
Plot of the effect of the interaction between main economic activity and group on the change in depressive symptoms between the pre-intervention time point and 12-month follow-up.

**Figure 5:**
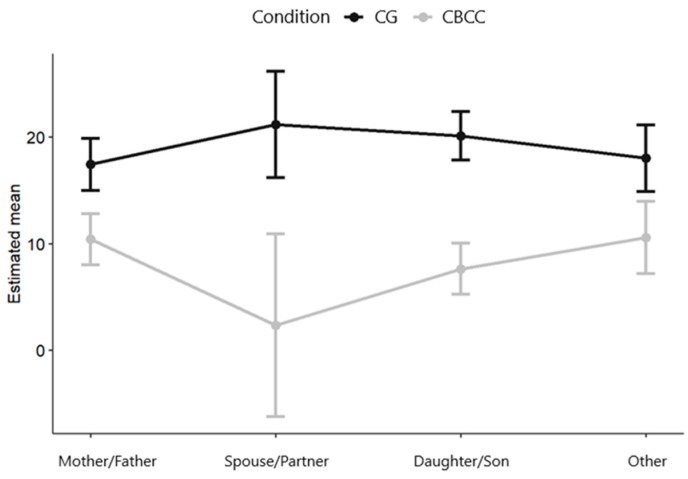
Plot of the effect of the interaction between relationship and group on the change in depressive symptoms between the pre-intervention time point and 12-month follow-up.

In the CBCC group, two variables moderating the effect of the intervention on the change in depressive symptoms were detected between the pre-intervention time point and the 12-month follow-up: main economic activity and relationship. Caregivers who were active in the workforce were more likely to benefit from the intervention than those who did not have paid jobs or who were retired (β = 6.41, *p* = 0.019, 95% CI = 1.06–11.76; see Figure 4). Likewise, those who cared for their partner (β = −11.85, *p* = 0.028, 95% CI = −22.43–−1.27) or their children (β = −5.467, *p* = 0.024, CI 95% = −10.193–−0.741) benefited the most from the intervention; see Figure 5.

For the BACC group, no moderators of the change in depressive symptoms were found between the pre-intervention time point and the 12-month follow-up.

Table 5 shows the results of the regression equations for the mediation models for the CBCC group. The change in positive environmental reinforcement (pre-intervention to post-intervention) and in negative automatic thoughts (pre-intervention to post-intervention) acted as partial mediators of the difference in depressive symptoms (pre-intervention to post-intervention), with a significant mediation effect of −2.89 (95% CI −4.91–−1.28) for positive environmental reinforcement and −1.21 (95% CI −2.42–−0.25) for negative automatic thoughts. The mediating effect of change in positive environmental reinforcement explained 38.6% of the total effect of the treatment on changes in depressive symptoms. The mediating effect of change in negative automatic thoughts explained 15.7% of the total effect of the intervention on changes in depressive symptoms. Social contacts did not show a significant mediating effect.

Table 6 shows the results of the regression equations for the mediation models for the BACC group. The change in positive environmental reinforcement (pre-intervention to post-intervention) and in negative automatic thoughts (pre-intervention to post-intervention) acted as partial mediators of the difference in depressive symptoms (pre-intervention to post-intervention), with a significant mediation effect of −2.52 (95% CI −4.18–−1.27) for positive environmental reinforcement and −1.02 (95% CI −2.16–−0.15) for negative automatic thoughts. The mediating effect of change in positive environmental reinforcement explained 29% of the total effect of the treatment on changes in depressive symptoms. The mediating effect of change in negative automatic thoughts explained 11.5% of the total effect of the intervention on changes in depressive symptoms. Social contacts did not show a significant mediating effect.

## 4. Discussion

The main objective of this study was to evaluate the long-term efficacy of a complete cognitive–behavioral intervention versus the behavioral-activation component only, administered via conference call, for the indicated prevention of depression in non-professional caregivers. The results indicate that the preventive effects were maintained for up to 36 months after the end of the intervention for both intervention groups.

### 4.1. Primary Objective: Depressive Symptoms and Episodes

A significant reduction in depressive symptoms was found in both the CBCC and BACC groups with respect to the CG, an effect maintained at the 36-month follow-up, with large effect sizes (CBCC *d* = 1.06 and BACC *d* = 0.93); there were no differences between the two intervention groups. These results are similar to those of other studies that also evaluated indicated depression prevention interventions (delivered in person) for caregivers. Specifically, the effect size for the CBCC group was similar to that achieved at 12 months with the same cognitive–behavioral intervention administered in person (*d* = 1.33; [21]), as well as that found at 12 months (*d* = 1.14) in another study evaluating a face-to-face problem-solving intervention [20]. It was higher than the 0.39 effect size found in that same intervention [20] after 8 years of follow-up [19]. 

To our knowledge, there are no studies that have conducted follow-up of telephone interventions for caregivers with a duration equal to or greater than three years. The longest follow-up that we know of—two years [35]—did not find significant differences in depressive symptoms between a cognitive–behavioral intervention group and a control group of usual care (although differences in well-being were present). A possible explanation for these contrasting results is that in this study, specific inclusion and exclusion criteria were established to select a sample with a homogeneous level of depressive symptoms; that is, participants had a high level of depressive symptoms and the absence of a major depressive episode. As a result, this study avoided a possible floor effect that could prevent finding improvements if the same intervention were administered to caregivers without depressive symptoms, with different levels of depressive symptoms, and with major depression [86]. Furthermore, these comparisons should be made with caution, due to the difference in the duration of follow-up between the studies. 

At the 36-month follow-up, there was a lower incidence of depression in the two intervention groups compared to the CG (8.7% in CBCC, 8.6% in BACC, and 33.7% in CG); the RR was 0.26 for CBCC and 0.25 for BACC. For both interventions, it was found that approximately one new case of depression would be prevented for every four caregivers treated. Follow-ups for previous telephone interventions aimed at caregivers [34,35,36,39] did not assess the incidence of depressive episodes. However, in this study the outcomes for the two interventions are similar to those found in the same comprehensive cognitive–behavioral intervention for indicated prevention of depression in caregivers administered face-to-face [21]. Vázquez et al. [21] found that the incidence at 12 months was 3.4% in the intervention group and 22.0% in the control group, with an RR of 0.15 and an NNT of 5. In addition, our previous work examining a problem-solving intervention for the indicated prevention of depression administered face-to-face found that the incidence of major depression at 12 months was 10.1% for the intervention group versus 25.0% in the control group, with an RR of 0.40 and an NNT of 7 [20]. Furthermore, the incidence at the 8-year follow-up was 30.3% in the intervention group, compared to 26.2% in the control group [19]. However, given the difference in the length of follow-up, the data are possibly not directly comparable. 

Likewise, the incidence of depression in the current study was higher than that found in Cuijpers et al.’s [87] meta-analysis of psychotherapies for sub-clinical depression, which found an NNT of 5 at the post-intervention time point, and an RR of 0.74 at the 12-month follow-up. It are also higher than those in Van Zoonen et al.’s meta-analysis [14], which found an RR of 0.40 and an NNT of 13 for indicated depression prevention interventions. Furthermore, in the current study, there was a significant delay in the development time of depression in the intervention groups compared to the control group, with no difference between the two groups. These results indicate that the CBCC and BACC interventions are effective in preventing (or at least delaying) new cases of depression. 

Moreover, the absence of significant differences between the two intervention groups in this study reveals that even 36 months after receiving the intervention, the behavioral activation component alone continued to be as effective as the complete cognitive–behavioral intervention in reducing depressive symptoms. This finding is consistent with the Multifactorial Integrative Model of Depression by Lewinsohn, Hoberman, Teri and Hautzinger [53], which considers depression as the final result of changes initiated by the environment on the emotions, behaviors and thoughts, with consideration to the existence of feedback loops. This implies that an intervention on any of the variables in the model could work both by aggravating depression and improving it. It is also consistent with previous evidence that behavioral activation is as effective as a comprehensive cognitive–behavioral intervention in the indicated prevention of short-term depression [40] and in the treatment of clinical depression at up to 6 months of follow-up [88]. However, it is the first such study that demonstrated the long-term (i.e., 36 months) duration of this effect.

No differences were found among the three groups in the percentage of caregivers who had experienced the death of the care recipient, and the results after controlling for this covariate were very similar to those in the unadjusted models. This suggests that the death of the relative did not influence the results and is consistent with the findings from previous studies (e.g., [19]).

### 4.2. Secondary Objective: Moderators and Mediators

The secondary objective of this study was to examine the moderators and mediators of the effects of the interventions. The CBCC group intervention was more beneficial for those caregivers who were active in the workforce and those who cared for their partner or their children. No moderators were found for the BACC group intervention. The finding that the results were better for those who were active in the workforce are consistent with Alcántara et al.’s [89] study of a cognitive–behavioral concern-reduction intervention for treating depression, which found that workers in paid employment showed the greatest decreases in concern for all experimental conditions. However, our results contrast with a previous study that found in two indicated prevention interventions for caregivers that at the 12-month follow-up, the risk of depression for those with intermediate emotional distress was higher for those caregivers who worked outside the home [90]. One possible explanation is that caregivers who combine care tasks with work tasks benefit from negative thought reduction strategies as a way to also manage negative thoughts related to their work activities, which would lead to a decrease in negative affect in various areas of their life. 

The current study also found that greater benefits were obtained by those who cared for their partner or their children. These results are partially consistent with those by Kim, Zarit, Femia, and Savla [91], who found that both the partner and daughters of people who participated in the intervention had better results in relation to depressive symptoms compared to controls. However, they are inconsistent with Sörensen, Pinquart, and Duberstein [92], who found that those who cared for their partners had worse results from the interventions than those who cared for their parents. The finding that there were no moderating variables for the BACC group suggests that this intervention has comparable efficacy among a wide range of demographic groups and in different care settings.

Finally, the current study found that the change in positive environmental reinforcement and negative automatic thoughts between the pre- and post-intervention time points facilitated the reduction of depressive symptoms in both the CBCC and BACC groups, with greater weight on positive environmental reinforcement for both intervention groups. These findings are partially consistent with the mediating role of the change in negative thoughts found in the same cognitive–behavioral intervention when administered face-to-face [21]. Together, this suggests the usefulness of both the behavioral and cognitive techniques of the intervention (with greater weight on the former). However, these particular findings are contrary to what was found in Jacobson et al.’s study [45], where an initial change in attributional style at early points was associated with a change in depressive symptoms for the behavioral-activation only group (but not for the complete cognitive–behavioral intervention group). Furthermore, change in the frequency of enjoyable activities was associated with change in the complete cognitive–behavioral intervention group, but not in the behavioral-activation group. The results for automatic thoughts mediated change in the BACC group might be explained by applying the Interactive Cognitive Subsystem Model [93,94], which explains that lived experiences—unlike the more rational cognitive techniques, which follow slow and logical information processing—tend to impact information processing at a different level when associated with high levels of emotion, and therefore they have rapid and powerful effects in modifying depressive cognitive patterns.

### 4.3. Strengths and Limitations

This work has some important strengths. To our knowledge, this is the first study to conduct a 36-month follow-up on an indicated depression prevention intervention aimed at caregivers using a telephone format, as well as the first in which a dismantling strategy has been used in a study aimed at caregivers. It provides evidence that both a cognitive–behavioral conference call intervention and an intervention that included only the behavioral-activation component resulted in significant differences in the incidence of depression and depressive symptoms at all follow-up points through 36 months when compared to a control group of usual care. This underscores the efficacy of telephone formats for the indicated prevention of depression in this population long term. It also shows that both interventions yield similar results in the reduction of depressive symptoms and the incidence of depression, a finding that provides information on the necessary and sufficient components to achieve therapeutic change. At the methodological level, both interventions were based on a well-founded theoretical model, and standardized and reliable instruments were used to evaluate the results. 

However, it is also necessary to point out some limitations. Most of the sample in this study consisted of women, which could make it difficult to generalize their results to male caregivers. However, this limitation is minimal, considering that the majority of caregivers are women [95,96]. In addition, the evaluations were conducted over the phone, which may result in missing visual cues that could be clinically relevant to diagnosis [97]. However, previous works have found that interviews using this method achieve results comparable to those from face-to-face evaluations [98]. Finally, this study was carried out in Spain, and its findings may not be generalizable to other countries. Studies on the long-term efficacy of indicated depression prevention in different cultural settings are needed.

### 4.4. Implications for Research and Clinical Practice

This study has important implications for research and clinical practice. It provides evidence that two telephone interventions to prevent depression in caregivers offer long-term efficacy and that an intervention focused on the more parsimonious behavioral-activation component achieves long-term results comparable to those of the complete multicomponent cognitive–behavior intervention. This represents an important advance in the refinement of preventive programs because it makes it possible to have an intervention with equally optimal and long-lasting effectiveness that is more accessible as a result of the conference call format. Due to restrictions on time and alternative care for the care recipient, telephone interventions are likely to be more accessible for caregivers and clinicians. In addition, these interventions can be administered by specialized personnel after brief training. Furthermore, this study suggests that a comprehensive cognitive–behavioral intervention is particularly beneficial for caregivers in paid employment and for those who care for their partner or children. Meanwhile, the intervention containing only the behavioral-activation component was equally beneficial for all caregiver profiles. Taking moderators into account during clinical decision-making could help in choosing more personalized and effective treatments. Finally, this study provides evidence of the mechanisms of change underlying the therapeutic effects of the preventive intervention.

## 5. Conclusions

This study provides evidence of the long-term efficacy of a cognitive–behavioral intervention and an intervention that included only the behavioral activation component. These interventions were administered by conference call to non-professional caregivers, supporting the use of telephone formats of administration aimed at the targeted prevention of depression in this population. The two interventions reduced the incidence and symptoms of depression compared to a usual care control group at the 36-month follow-up. No differences were found between the two interventions, demonstrating that the behavioral activation component is a central element of cognitive–behavioral interventions. This lays the foundation for the development of simple but highly efficacious programs for the targeted prevention of depression in the caregiver population. Furthermore, the finding that change in positive environmental reinforcement and negative automatic thoughts mediated the reduction of depressive symptoms between the pre- and post-intervention time points provides further support for the importance of these components in interventions for therapeutic change.

## Figures and Tables

**Table 1:** Contents of the indicated depression prevention interventions administered by telephone conference call.

Session	CBCC	BACC
Session 1	Presentation Purpose of the program Information about depression and active coping with symptoms Activation control training (diaphragmatic breathing) Monitoring mood Self-reinforcement Intersessional tasks	Presentation Purpose of the program Information about depression and active coping with symptoms Monitoring mood Self-reinforcement Intersessional tasks
Session 2	Explanation of the relationship between activities and mood Guidelines and strategies to increase enjoyable activities Planning enjoyable activities Behavioral contract Intersessional tasks	Explanation of the relationship between activities and mood Guidelines and strategies to increase enjoyable activities Planning enjoyable activities at home Behavioral contract Intersessional tasks
Session 3	Explanation of the relationship between thoughts and mood Techniques for managing thoughts Planning enjoyable activities Behavioral contract Intersessional tasks	Review of the relationship between enjoyable activities and mood Guidelines and strategies to increase enjoyable activities outside the home Planning enjoyable activities away from home Behavioral contract Intersessional tasks
Session 4	Explanation of the relationship between social contacts and mood Guidelines and strategies to increase and improve social relationships Planning of enjoyable social activities Behavioral contract Intersessional tasks	Explanation of the relationship between social contacts and mood Guidelines and strategies to increase social relationships Planning of enjoyable social activities Behavioral contract Intersessional tasks
Session 5	Review of everything learned Maintaining progress Relapse prevention Farewell and closure	Review of everything learned Maintaining progress Relapse prevention Farewell and closure

Note: CBCC = Cognitive–behavioral conference call intervention; BACC = Behavioral activation conference call intervention.

**Table 2:** Sociodemographics, clinical characteristics, and care situation of the study participants.

Characteristics	Total (*n* = 219)	CBCC (*n* = 69)	BACC (*n* = 70)	CG (*n* = 80)
Sex, *n* (%)				
Male	20 (9.1)	7 (10.1)	3 (4.3)	10 (12.5)
Female	199 (90.9)	62 (89.9)	67 (95.7)	70 (87.5)
Mean age (SD)	54.0 (10.8)	54.8 (10.7)	54.5 (11.0)	52.9 (10.7)
Marital status, *n* (%)				
Single	25 (11.4)	7 (10.2)	9 (12.9)	9 (11.2)
Married, lives as a couple	157 (71.7)	51 (73.9)	52 (74.2)	54 (67.5)
Separated/Divorced/Widowed	37 (16.9)	11 (15.9)	9 (12.9)	17 (21.3)
Social class, *n* (%)				
Low/Lower middle	114 (52.1)	36 (52.2)	39 (55.7)	39 (48.7)
Middle/Upper middle	105 (47.9)	33 (47.8)	31 (44.3)	41 (51.3)
Level of education, *n* (%)				
Can read and write	27 (12.3)	5 (7.3)	12 (17.1)	10 (12.5)
Primary	123 (56.2)	39 (56.5)	38 (54.3)	46 (57.5)
Secondary/University	69 (31.5)	25 (36.2)	20 (28.6)	24 (30.0)
Main economic activity, *n* (%)				
Active in the workforce	46 (21.0)	11 (15.9)	16 (22.9)	19 (23.7)
No paid employment/Retired	173 (79.0)	58 (84.1)	54 (77.1)	61 (76.3)
Care recipient sex, *n* (%)				
Male	85 (38.8)	28 (40.6)	28 (40.0)	29 (36.2)
Female	134 (61.2)	41 (59.4)	42 (60.0)	51 (63.8)
Care recipient age (SD)	60.8 (33.1)	59.9 (32.7)	67.6 (30.0)	55.5 (35.2)
Relationship with care recipient, *n* (%) Providing care for their:				
Father/mother	86 (39.3)	27 (39.1)	32 (45.7)	27 (33.7)
Spouse/partner	12 (5.5)	2 (3.0)	4 (5.7)	6 (7.5)
Child	75 (34.2)	27 (39.1)	17 (24.3)	31 (38.8)
Others	46 (21.0)	13 (18.8)	17 (24.3)	16 (20.0)
Care recipient diagnosis, *n* (%)				
Diseases of the musculoskeletal system, connective tissue, cardiovascular and respiratory systems	53 (24.2)	13 (18.8)	18 (25.7)	22 (27.5)
Chromosomal, congenital, and perinatal abnormalities	39 (17.8)	11 (15.9)	13 (18.6)	15 (18.8)
Mental disorders, neurological diseases, and brain damage	62 (28.3)	21 (30.5)	16 (22.8)	25 (31.2)
Dementias	65 (29.7)	24 (34.8)	23 (32.9)	18 (22.5)
Duration of care provision (SD)	12.8 (9.1)	13.9 (9.8)	12.8 (9.0)	11.9 (8.5)
Daily hours of care (SD)	15.8 (4.1)	15.3 (4.4)	16.2 (3.8)	15.9 (4.0)
Depressive symptomatology (SD)	22.7 (6.3)	22.3 (6.2)	22.7 (6.8)	23.1 (5.9)
Positive environmental reinforcement (SD)	26.8 (4.4)	25.9 (4.1)	27.2 (4.4)	27.2 (4.6)
Automatic negative thoughts (SD)	50.7 (16.5)	51.4 (15.9)	50.1 (17.0)	50.5 (16.7)
Social contacts (SD)	22.0 (18.0)	20.9 (13.8)	25.2 (23.5)	20.1 (15.3)

Note: CBCC: Cognitive–behavioral conference call intervention; BACC: Behavioral activation conference call intervention; CG: Usual care control group.

**Table 3:** Estimated mean scores (and standard errors) for depressive symptoms for the intervention groups and the control group.

Time Point	CBCC (*n* = 69)	BACC (*n* = 70)	CG (*n* = 80)
Pre-intervention	22.3 (0.9)	22.7 (0.9)	23.1 (0.8)
Post-intervention	11.3 (0.9)	10.4 (0.9)	19.6 (0.8)
1-month follow-up	8.6 (0.9)	8.3 (0.9)	19.1 (0.8)
3-month follow-up	10.1 (0.9)	9.2 (0.9)	18.4 (0.8)
6-month follow-up	8.8 (0.9)	10.2 (0.9)	19.1 (0.9)
12-month follow-up	9.0 (0.9)	10.5 (0.9)	19.0 (0.9)
36-month follow-up	9.3 (1.0)	10.3 (0.9)	17.3 (1.0)

**Table 4:** Student’s *t* statistics and standardized mean differences for intragroup and intergroup effects.

Comparison	*t*	*p*	*d*	95% CI
**Intragroup effects tests**				
*CBCC Group*				
Pre-intervention/Post-intervention	11.524	<0.001	1.46	1.20–1.71
Pre-intervention/1-month follow-up	14.432	<0.001	1.83	1.57–2.08
Pre-intervention/3-month follow-up	12.842	<0.001	1.63	1.37–1.88
Pre-intervention/6-month follow-up	14.044	<0.001	1.79	1.53–2.05
Pre-intervention/12-month follow-up	13.953	<0.001	1.77	1.51–2.03
Pre-intervention/36-month follow-up	12.536	<0.001	1.73	1.45–2.01
*BACC Group*				
Pre-intervention/Post-intervention	12.795	<0.001	1.64	1.38–1.89
Pre-intervention/1-month follow-up	15.001	<0.001	1.91	1.66–2.17
Pre-intervention/3-month follow-up	14.054	<0.001	1.80	1.54–2.05
Pre-intervention/6-month follow-up	13.103	<0.001	1.67	1.41–1.92
Pre-intervention/12-month follow-up	12.745	<0.001	1.62	1.36–1.87
Pre-intervention/36-month follow-up	12.719	<0.001	1.65	1.39–1.91
*Group CG*				
Pre-intervention/Post-intervention	3.981	<0.001	0.47	0.24–0.70
Pre-intervention/1-month follow-up	4.573	<0.001	0.54	0.31–0.77
Pre-intervention/3-month follow-up	5.356	<0.001	0.63	0.40–0.86
Pre-intervention/6-month follow-up	4.571	<0.001	0.54	0.31–0.77
Pre-intervention/12-month follow-up	4.619	<0.001	0.56	0.32–0.79
Pre-intervention/36-month follow-up	5.899	<0.001	0.78	0.52–1.04
**Intergroup effects tests**				
* Post-intervention*				
CBCC/CG Group	6.62	<0.001	1.10	0.77–1.43
BACC/CG Group	7.349	<0.001	1.23	0.90–1.56
* 1-month follow-up*				
CBCC/CG Group	8.434	<0.001	1.40	1.07–1.73
BACC/CG Group	8.597	<0.001	1.44	1.11–1.77
* 3-month follow-up*				
CBCC/CG Group	6.684	<0.001	1.11	0.78–1.44
BACC/CG Group	7.332	<0.001	1.23	0.89–1.56
* 6-month follow-up*				
CBCC/CG Group	8.186	<0.001	1.37	1.04–1.70
BACC/CG Group	7.136	<0.001	1.19	0.86–1.51
* 12-month follow-up*				
CBCC/CG Group	7.882	<0.001	1.33	0.99–1.66
BACC/CG Group	6.649	<0.001	1.12	0.79–1.46
* 36-month follow-up*				
CBCC/CG Group	5.59	<0.001	1.06	0.69–1.44
BACC/CG Group	5.24	<0.001	0.93	0.58–1.28

Note: Only the following showed significant results: *p* = *p* adjusted for Bonferroni, Holm–Bonferroni, and Benjamini and Yekutieli corrections (all *p* were equal); *d* = effect size (Cohen’s *d*), CI = Confidence Interval; CBCC = cognitive–behavioral conference call intervention; BACC = behavioral-activation conference call intervention; CG = usual care control group.

**Table 5:** Analysis of mediation for positive environmental reinforcement, negative automatic thoughts, and social contacts at the post-intervention time point for the CBCC group.

Parameter		Estimated (Weighted) Coefficient	95% CI	*t*	*p*	DF	RIV	RE
Y = X								
	c	−7.52	−10.28–−4.76	−5.39	<0.001	144.15	0.006	0.9987
M = X								
Environmental reinforcement	a	1.08	0.80–1.37	7.45	<0.001	143.91	0.007	0.9986
Automatic negative thoughts	a	−0.37	−0.65–−0.08	−2.57	0.011	143.84	0.008	0.9986
Social contacts	a	0.16	−0.11–0.43	1.16	0.249	144.11	0.006	0.9987
Y = X + M								
Environmental reinforcement	c′	−4.63	−7.76–−1.51	−2.93	0.0040	143.04	0.007	0.9987
								
Environmental reinforcement	b	−2.67	−4.19–−1.15	−3.47	0.001	141.83	0.014	0.9982
Automatic negative thoughts	c′	−6.31	−8.98–−3.65	−4.68	<0.001	143.32	0.005	0.9988
Automatic negative thoughts	b	3.32	1.79–4.84	4.30	<0.001	141.62	0.015	0.9981
Social contacts	c′	−7.30	−10.06–−4.54	−5.24	<0.001	143.11	0.006	0.9987
Social contacts	b	−1.41	−3.07–0.25	−1.68	0.095	141.65	0.015	0.9981

Note: 95% CI = 95% confidence interval; DF = degrees of freedom; RIV = relative increase in variance; RE = relative efficiency.

**Table 6:** Analysis of mediation for positive environmental reinforcement, negative automatic thoughts, and social contacts at the post-intervention time point for the BACC group.

Parameter		Estimated (Weighted) Coefficient	95% CI	*t*	*p*	DF	RIV	RE
Y = X								
	c	−8.75	−11.45–−6.05	−6.40	<0.001	142.67	0.020	0.9978
M = X								
Environmental reinforcement	a	0.70	0.42–0.98	4.92	<0.001	141.80	0.024	0.9975
Automatic negative thoughts	a	−0.40	−0.75–−0.04	−2.23	0.027	144.27	0.011	0.9984
Social contacts	a	−0.09	−0.43–0.24	−0.55	0.582	144.48	0.010	0.9985
Y = X + M								
Environmental reinforcement	c′	−6.23	−8.94–−3.52	−4.54	<0.001	141.95	0.018	0.9979
Environmental reinforcement	b	−3.59	−5.06–−2.12	−4.82	<0.001	139.22	0.032	0.9970
Automatic negative thoughts	c′	−7.73	−10.32–−5.13	−5.89	<0.001	141.50	0.021	0.9977
Automatic negative thoughts	b	2.59	1.39–3.79	4.28	<0.001	138.57	0.035	0.9969
Social contacts	c′	−8.84	−11.53–−6.14	−6.48	<0.001	141.55	0.021	0.9978
Social contacts	b	−0.92	−2.23–0.38	−1.40	0.163	138.96	0.033	0.9970

Note: 95% CI = 95% confidence interval; DF = degrees of freedom; RIV = relative increase in variance; RE = relative efficiency.
